# Supplementary material for: Treatment with IgM‐enriched intravenous immunoglobulins enhances clearance of stroke‐associated bacterial lung infection
Source: Immunology. 2022 Aug 9;167(4):558–75. doi: 10.1111/imm.13553 (PMC11495265; doi:10.1111/imm.13553)
Supplement: Supplementary file 1 — Data S1 Supporting information. [file IMM-167-558-s001.docx]

| **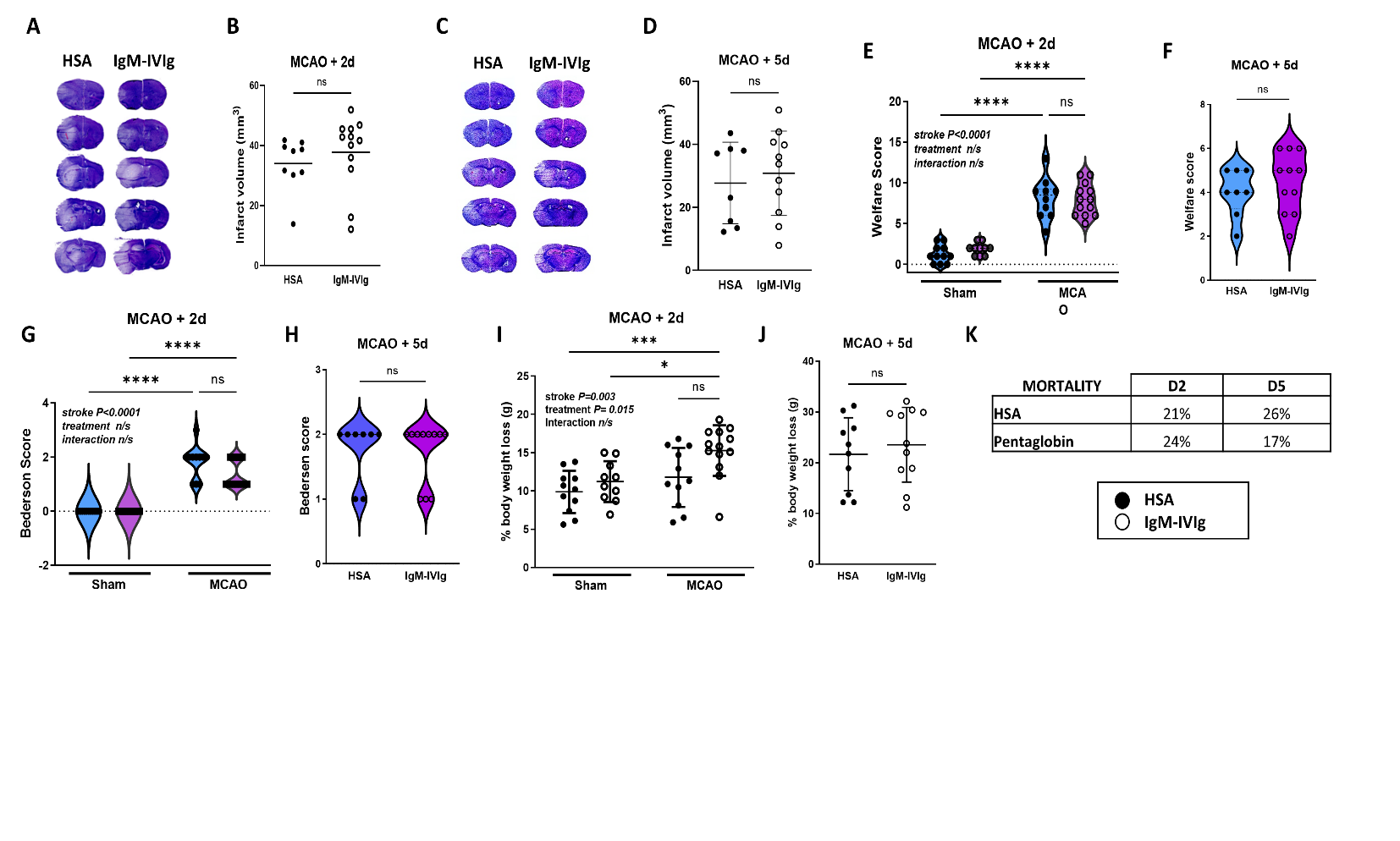** |
| --- |
| **Supplementary Figure 1 Low dose IgM-IVIg does not modulate primary stroke pathology**  Infarct volume (mm^3^) in the brains of animals treated with human serum albumin (HSA; ●) or IgM-IVIg (○) at (**A, B**) 2 days or (**C, D**) 5 days post MCAO. Treatment with IgM-IVIg did not affect welfare score at (**E**) 2 days or (**F**) 5 days after MCAO. (**G**) Animals treated with IgM-IVIg had a higher percentage reduction in bodyweight 2 days post sham or MCAO surgery. (**H**) However at 5 days post MCAO there was no effect of treatment with IgM-IVIg on percentage body weight loss. Treatment with IgM-IVIg did not affect Bedersen score at (**I**) 2 days or (**J**) 5 days after MCAO. (**K**) Treatment with IgM-IVIg had no effect on mortality at 2 days after MCAO however at 5 days post MCAO, mortality was lower in mice treated with IgM-IVIG (**B, D, G, H, K, L**) Data show data points with mean ± S.D; * P<0.05; ** P<0.01; *** P<0.001; P<0.0001; (**B, D, H, L**) unpaired t-test. (**G, K**) two way ANOVA with Tukey’s multiple comparison test; (**E, F, I, J**) Violin plots with Mann-Whitney   \|  \| **Score** \| **Criteria** \| \| --- \| --- \| --- \| \| **Coat appearance** \| 0 \| Normal \| \|  \| 1 \| Piloerection \| \|  \| 2 \| Severe piloerection \| \| **Grooming** \| 0 \| Normal \| \|  \| 1a \| Mild impairment \| \|  \| 1b \| Excessive \| \|  \| 2 \| None \| \| **Ocular/ Nasal discharge** \| 0 \| None \| \|  \| 1 \| Discharge present \| \| **Posture** \| 0 \| Normal \| \|  \| 1 \| Mild hunching \| \|  \| 2 \| Severe hunching \| \| **Vocalising** \| 0 \| Normal \| \|  \| 1 \| Mild/ intermittent \| \|  \| 2 \| Persistent/ distressed \| \| **Respiratory Rate** \| 0 \| Normal \| \|  \| 1 \| Mildly depressed \| \|  \| 2 \| Severely depressed \| \| **General exploratory** \| 0 \| Normal \| \| **behaviour** \| 1a \| Subdued \| \|  \| 1b \| Excessive \| \|  \| 2 \| No peer interaction/ exploration \| \| **Neurological Deficit** \| 0 \| Normal \| \| **(Bederson Score)** \| 1 \| Torso flexion upon tail lift \| \|  \| 2 \| Circling \| \|  \| 3 \| Severe circling/ leaning \| \|  \| 4 \| No movement \| \| **Responsiveness** \| 0 \| Spontaneous movement \| \|  \| 1 \| Responsive to touch \| \|  \| 2 \| Unresponsive \| \| **Tremors** \| 0 \| None \| \|  \| 1 \| Intermittent \| \|  \| 2 \| Persistent \| \| **Convulsions/ Seizure activity** \| 0 \| No \| \|  \| 1 \| Yes \| \| **Barrel-rolling** \| 0 \| No \| \|  \| 1 \| Yes \|   **Supplementary Table 1 Welfare scoring of animals post-surgery**  Scoring criteria to assess the welfare of animals as they recover from sham or MCAO surgery. The categories of “Responsiveness”. “Tremors”, “Convulsions/ Seizure activity” and “Barrel-rolling” reflect humane end points and animal that score in these categories would be culled for welfare.  **Supplementary Figure 2 Criteria for scoring lung pathology**  Scoring system used to grade the extent of pathology in lung from animals treated with HSA or IgM-IVIG 2 days and 5 days after sham or MCAO surgery (**Figure 1**). Goblet cell hyperplasia was not detected in any animals. All animals had <25% alveolar spaces with macrophages present and scored 1. Inflammation did not reach a score of 3 in any animals. Scores in sham-operated animals mainly consisted of edema and some with mild inflammation. Scale bars 50 mm.   \|  \| \| --- \| \| \| **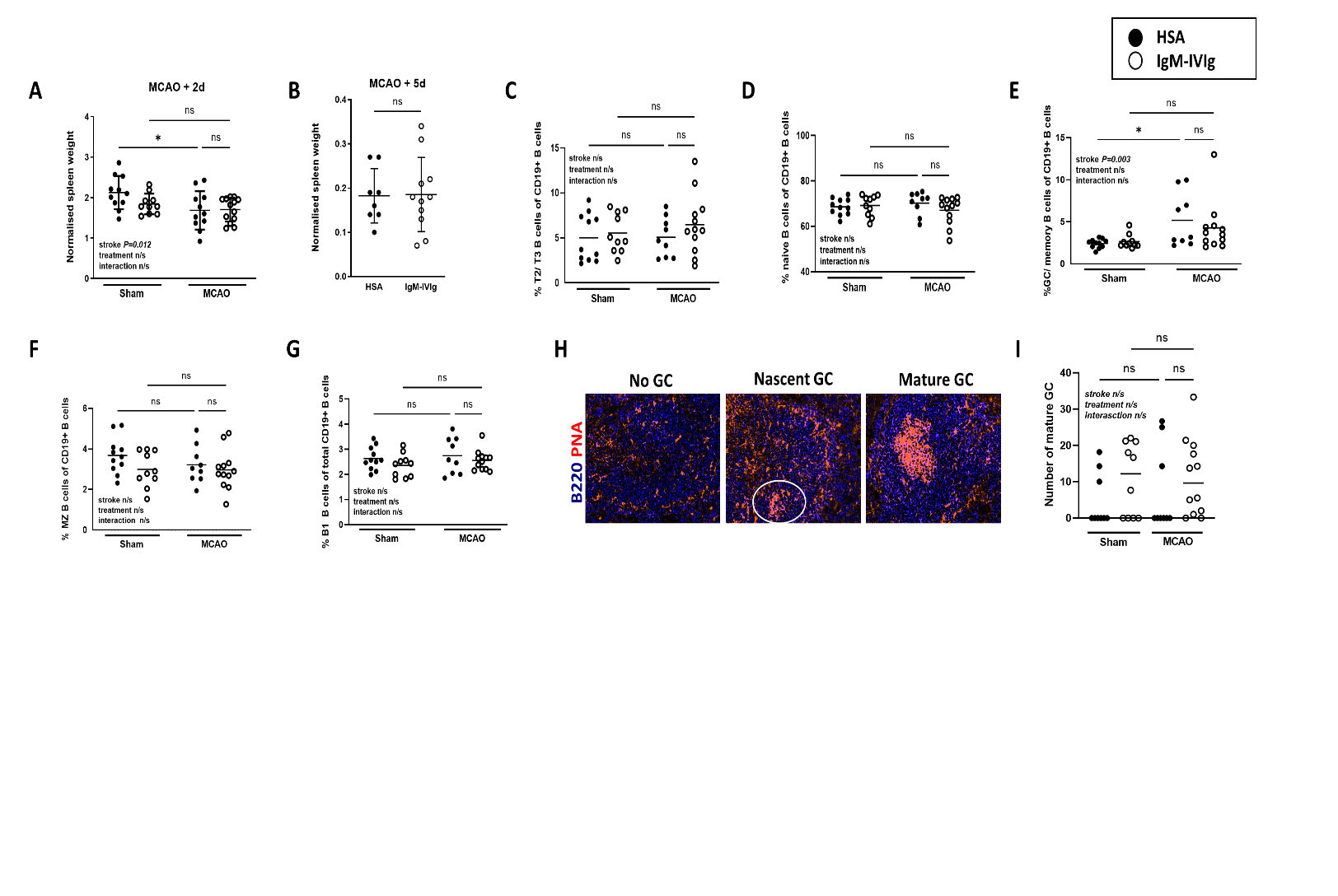** \| \| --- \| \| **Supplementary Figure 3 Splenic B cell responses to IgM-IVIg**  Spleen weight, normalised to total body weight, is significantly reduced by stroke (**A**) but there is no effect of treatment with IgM-IVIg at (**A**) 2 d or (**B**) 5 d after sham or MCAO surgery. Percentage of (**C**) IgD^+^CD23^+^CD24^+^CD93^+^ T2 transitional B cells (**D**) IgD^+^CD23^+^CD23^+^CD24^+^CD93^-^ naïve B cells (**E**) CD23^-^CD21^-^CD93^-^ GC and memory B cells (**F**) CD19^+^CD23^-^CD21^+^CD93^-^ marginal zone B cells and (**G**) CD93^-^CD43^+^ B1 B cells within the total CD19^+^ B cell population measured by flow cytometry of spleens from mice treated with human serum albumin (HSA; ●) or IgM-IVIg (○) and after 2 d recovery from sham or MCAO surgery. Gating strategy in Figure 3A (Sham HSA n=11; Sham IgM-IVIg n=10; MCAO HSA n=9; MCAO IgM-IVIg n=12). (**H**) Immunolabelling of germinal centres (GC) using peanut agglutinin (PNA; orange) and DAPI staining of nuclei (blue) in spleens to identify areas of white pulp with no GC, nascent GC and mature GC. (**I**) Number of mature GC per half spleen section in spleens from mice treated with human serum albumin (HSA; ●) or IgM-IVIg (○) and after 2 d recovery from sham or MCAO surgery (Sham HSA n=9; Sham IgM-IVIg n=10; MCAO HSA n=9; MCAO IgM-IVIg n=12). Data show data points with mean ± SD; (**A, B, C, D, E , G**) two way ANOVA with Tukey’s multiple comparison test \| \|  \| \| \| \| **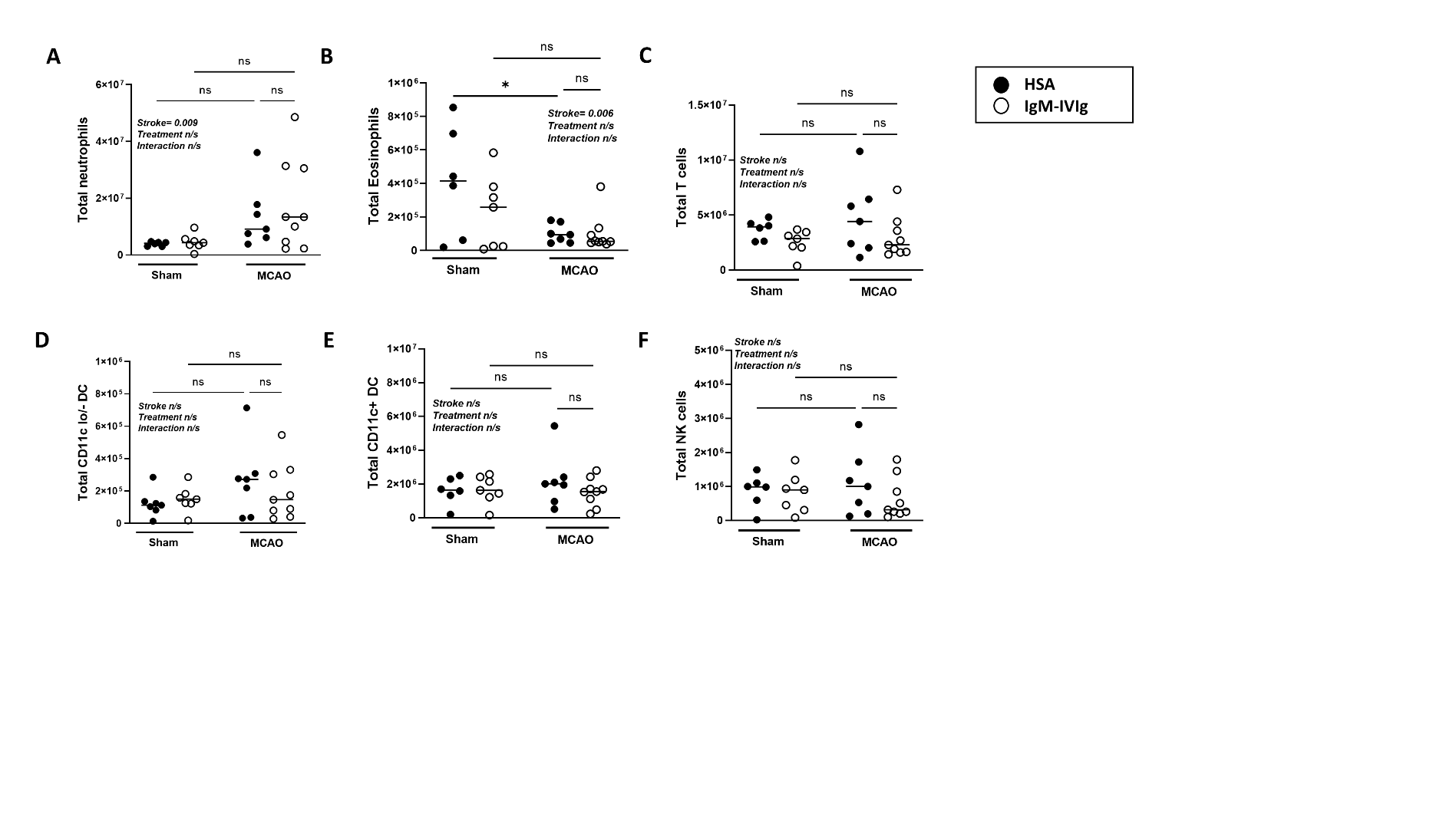** \| \| --- \| \| **Supplementary Figure 4 Effect of IgM-IVIg on lung immune cell subsets**  Total (**A)** CD11b+SiglecF-LygG^+^ neutrophils, (**B**) CD11b^+^SiglecF^+^LygG^-^ eosinophils, (**C**) CD3^+^CD20^-^ T cells, (**D**) CD11b^+^SiglecF^-^Ly6G^-^CD64^-^MHC II^+^ DC (**E**) CD11b ^+/-^CD11c^+^ DC and (**F**) CD11b^-^NKP46^+^ NK cells measured by flow cytometry of lung single cell suspensions from mice treated with human serum albumin (HSA; ●) or IgM-IVIg (○) and after 2 d recovery from sham or MCAO surgery. For gating strategy see Figure 4A. (Sham HSA n=6; Sham IgM-IVIg n=7; MCAO HSA n=7; MCAO IgM-IVIg n=10). Data show data points with mean ± S.D; * P<0.05; (**A-F**) two way ANOVA with Tukey’s multiple comparison test. \| \|  \| \| \|  \| \|  \| |
| \| **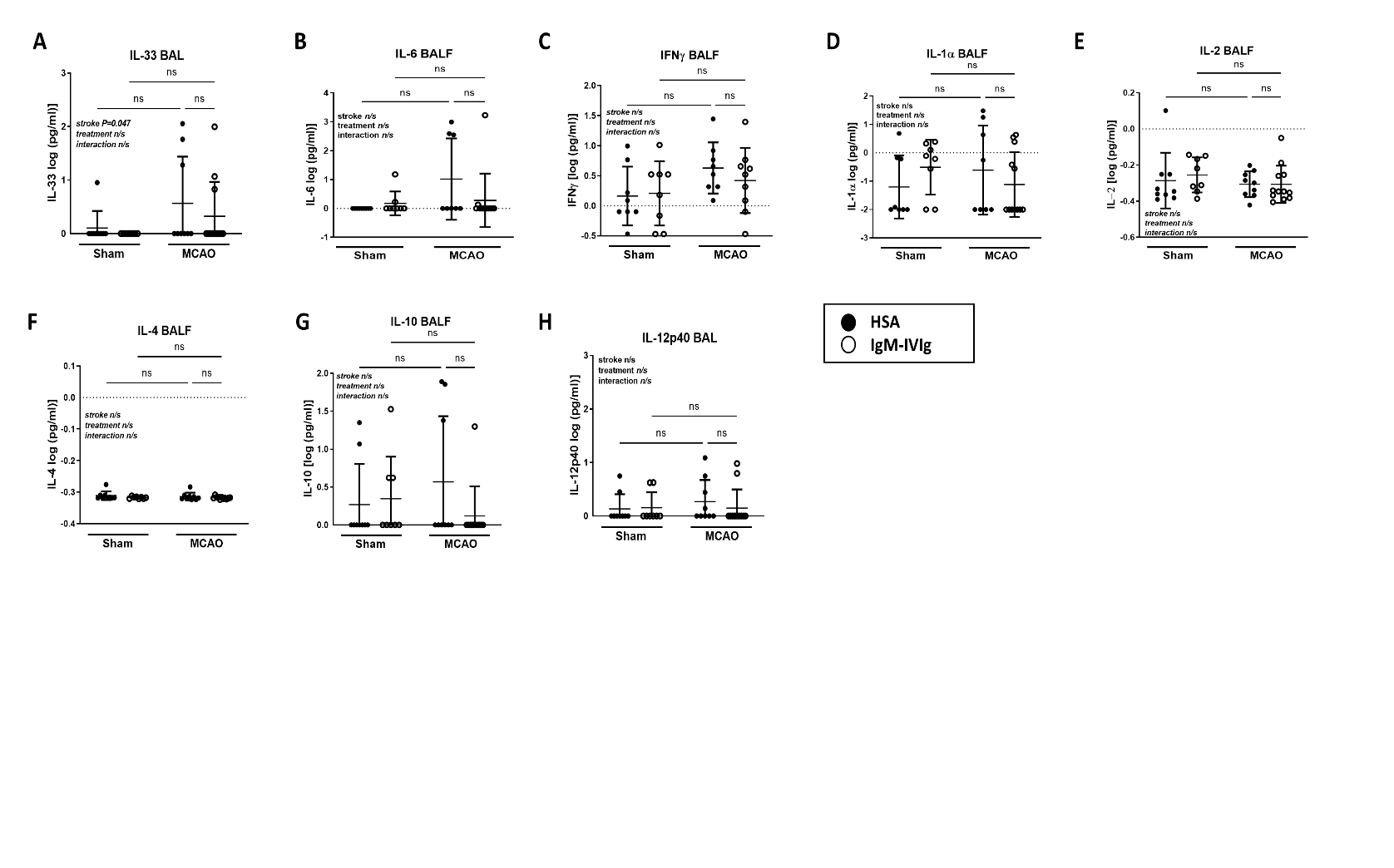** \| \| --- \| \| **Supplementary Figure 5 Effect of IgM-IVIg on lung immune cell subsets**  Concentration of (**A)** IL-1a (**B**) IL-2 (**C**) IL-4 (**D**) IL-6 (**E**) IL-10 (**F**) IL-12p40 **(G**) IL-33 and (**H**) IFNg measured by multiplexed ELISA of BALF from mice treated with human serum albumin (HSA; ●) or IgM-IVIg (○) and after 2 d recovery from sham or MCAO surgery (Sham HSA n=9; Sham IgM-IVIg n=8; MCAO HSA n=8; MCAO IgM-IVIg n=11). Data show data points with mean ± S.D; (**A-H)** two way ANOVA with Tukey’s multiple comparison test. \| \|  \| |
|  |
