# Supplementary figures and images for: Treatment with IgM‐enriched intravenous immunoglobulins enhances clearance of stroke‐associated bacterial lung infection
Source: Immunology. 2022 Aug 9;167(4):558–75. doi: 10.1111/imm.13553 (PMC11495265; doi:10.1111/imm.13553)

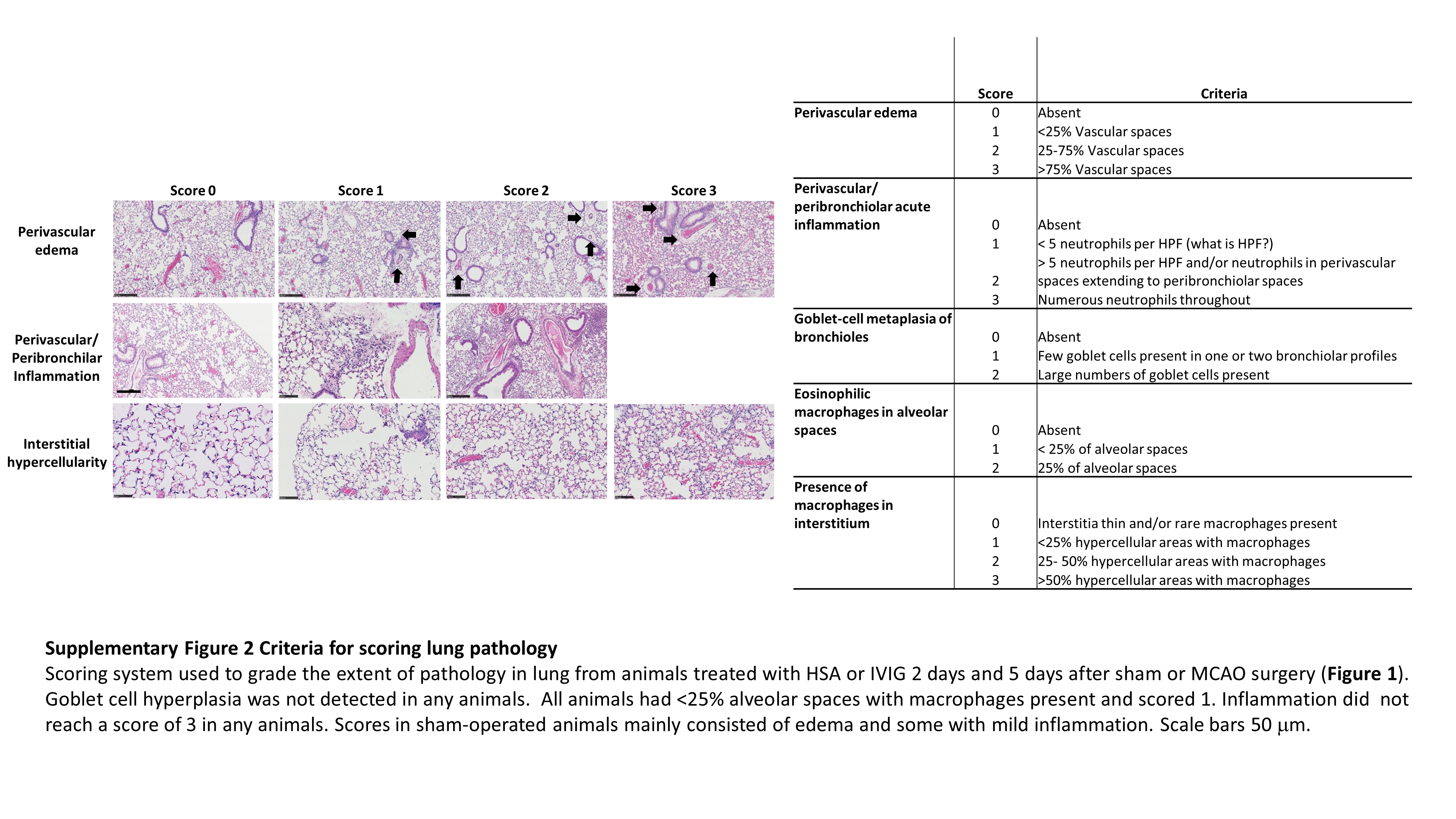

Supplement: Supplementary file 2 — Figure S2 Criteria for scoring lung pathology Scoring system used to grade the extent of pathology in lung from animals treated with HSA or IgM‐IVIG 2 days and 5 days after sham or MCAO surgery (Figure 1). Goblet cell hyperplasia was not detected in any animals. All animals had <25% alveolar spaces with macrophages present and scored 1. Inflammation did not reach a score of 3 in any animals. Scores in sham‐operated animals mainly consisted of oedema and some with mild inflammation. Scale bars 50 mm. [file IMM-167-558-s002.tif]
